# On the Choice of Access Point Selection Criterion and Other Position Estimation Characteristics for WLAN-Based Indoor Positioning

**DOI:** 10.3390/s16050737

**Published:** 2016-05-20

**Authors:** Elina Laitinen, Elena Simona Lohan

**Affiliations:** Department of Electronics and Communications Engineering, Tampere University of Technology, Korkeakoulunkatu 3, Tampere 33720, Finland; elena-simona.lohan@tut.fi

**Keywords:** indoor positioning, access point selection, received signal strength, fingerprinting, path loss, weighted centroid

## Abstract

The positioning based on Wireless Local Area Networks (WLAN) is one of the most promising technologies for indoor location-based services, generally using the information carried by Received Signal Strengths (RSS). One challenge, however, is the huge amount of data in the radiomap database due to the enormous number of hearable Access Points (AP) that could make the positioning system very complex. This paper concentrates on WLAN-based indoor location by comparing fingerprinting, path loss and weighted centroid based positioning approaches in terms of complexity and performance and studying the effects of grid size and AP reduction with several choices for appropriate selection criterion. All results are based on real field measurements in three multi-floor buildings. We validate our earlier findings concerning several different AP selection criteria and conclude that the best results are obtained with a maximum RSS-based criterion, which also proved to be the most consistent among the different investigated approaches. We show that the weighted centroid based low-complexity method is very sensitive to AP reduction, while the path loss-based method is also very robust to high percentage removals. Indeed, for fingerprinting, 50% of the APs can be removed safely with a properly chosen removal criterion without increasing the positioning error much.

## 1. Introduction

Indoor positioning has gained considerable attention in the last ten years. Global Navigation Satellite Systems (GNSS) take care of the positioning process successfully outdoors but cannot offer an accurate location estimate indoors due to multipaths, Non-Line-of-Sight (NLOS) and signal attenuation [1,2,3]; and, therefore, many different indoor positioning systems have been proposed. Wireless Local Area Network (WLAN) infrastructures are widely available in both commercial and residential buildings, and, therefore, they offer practical and cost-effective possibilities for positioning. One positioning technology is based on time delays, e.g., Time-Of-Arrival (TOA) or Round-Trip-Time (RTT) of the received signals. Time-delay based methods, however, require network synchronization and exact delay measurements. In addition, the underlying physical layer features, such as multiple access schemes and modulation technologies, vary a lot between the WLANs on the market. Therefore, time delay-based positioning approaches for WLAN positioning are still not widespread. Another alternative is to utilize RSS or the Received Signal Strength Indicator (RSSI). The RSS(I) are very attractive and economical for the Location Service providers because of their availability in almost every wireless device and because they are easy to access from the Application Programming Interface (API) layer.

Typically, RSS-based positioning methods have two stages: in a first stage, an off-line training data collection is performed, and, in the second stage, the on-line estimation is done [1,4,5]. In the training phase, models and databases are built based on collected information about indoor environment. The training data, also called radiomap, can be collected in semi-automatic or automatic modes such as the crowd-sourcing. In the estimation phase that demands real-time processing, the unknown position of a mobile station (MS) is estimated based on the measured RSSs of the available APs and the radiomap information saved in the training phase. The full radiomap contains the (*x*,*y*,*z*)-coordinates of each fingerprint or grid point in the building, together with the Medium Access Control (MAC)-addresses for each hearable AP and the corresponding RSS. Now, the RSS radiomap can be used for the localization purposes via several ways: by matching the measured RSSs by the MS with the radiomap (fingerprinting method, FP) [6,7,8], by triangulation approaches, based on mapping the transmitter-to-receiver distance with information collected from the radiomap (e.g., from estimation based on path-loss (PL) models or other statistical models) [9,10], or by some low-complexity methods, like weighted centroid (WeiC) [11,12,13]. Other possible methods are, e.g., clustering [14,15,16] and spectral compression [17] based methods, but the focus in this paper is on the three most widespread methods, namely FP, PL and WeiC. In FP, the whole radiomap database needs to be transferred to the mobile in order to be able to calculate the position estimate via pattern matching algorithms. In path-loss-based positioning approaches, only AP locations and the path-loss model parameters are needed in the position calculation from the radiomap, and, therefore, we can transfer only a part of the training database to the mobile. WeiC needs only AP locations for the position estimate, leading to only small data transfer needs. Thus, PL and WeiC are highly suitable for mobile-centric solutions, while FP is usually good in network-centric positioning or for small-scale solutions. We remark that each of the described method demands the radiomap database to be collected and saved in the training phase, as well as updates to the database, if the building layout or AP infrastructure is changed. The only method that would not need the radiomap is the WeiC but only if the AP locations were known. This is usually not the case in reality, and, therefore, AP locations need to be estimated using the complete radiomap [10].

In many buildings, the AP infrastructure is very dense, leading to a huge amount of data for the positioning methods to deal with. The memory requirements for the fingerprint database in large areas or buildings may become overwhelming, and also data transmission may become a bottleneck for the positioning system, especially for fingerprinting. In addition, all available information is not needed for the positioning purposes at all. With such high deployment of APs in many buildings, it is clear that some APs are more relevant than others, and the unnecessary APs can be simply seen as noise [1,18]. This holds for, e.g., WLAN transmitters supporting multiple Basic Service Set Identifiers (BSSID), leading to situations where several MAC addresses can be seen at exactly the same location. Typically, the deployment of the AP transmitters inside a building is optimized primarily for communication goals, such as serving many users in the best possible way. This means that the location of several APs can be close to each other, transmitting more or less correlated information. Since RSS is dependent on the distance between the mobile and an AP, as well as on the topology of the environment, closely located APs may have heavily correlated RSSs. Thus, for positioning purposes, not all APs carry significant information and can be dropped from the estimation process, since redundant and unnecessary APs increase both the time and space complexity to build a positioning system [19]. By choosing only a subset of APs to be used in the positioning process, both the storage requirements and computational complexity can be decreased. In addition, with a properly chosen AP among the existing ones, we can not only diminish the amount of data to be stored and transferred, but we can also improve the location accuracy [20,21]. Besides AP reduction, the size of the radiomap can be cut down by decreasing the number of stored fingerprints. If the gathered measurements are mapped to fixed grids, the grid size can be *a* m × *a* m with a=1,5,10,20,etc., where bigger grid size decreases the size of the full database remarkably.

In our earlier work in [22], we studied several AP selection criteria with FP and PL positioning methods. This paper is an extension of the work in [22], and, in this paper, two main parameters are studied, namely the impact of grid size and the impact of AP selection, on three different positioning algorithms (FP, PL, and WeiC). In particular, when compared to [22], in this paper, all three algorithms are compared in terms of number of parameters needed to be saved and transmitted to the mobile, in terms of time consumption of the algorithm, as well as in terms of positioning accuracy, if parts of the data is removed or if the grid size is increased. Indeed, in this paper, we also investigate a new selection criterion. The purpose of our paper is to make the choice between different positioning algorithms and characteristics easier, since this kind of comprehensive studies are missing. AP selection has been previously studied also in [4,23,24,25,26,27,28,29,30], but grid size has not been addressed before in this concept. We will also summarize the findings of a suitable AP selection criteria shown in our previous publications [21,22]. The results are based on new datasets of real field measurements, collected with a Nexus tablet, in Tampere, Finland and in Berlin, Germany in two multi-floor office buildings and one multi-floor shopping mall. All three building scenarios include a large amount of gathered measurements. We have used in our data collection proprietary software solutions and HERE indoor building maps.

## 2. Related Work

In order to solve the challenges caused by a huge amount of available data, AP selection criteria have gained a lot of interest within the last few years [23,24,25,26,28,29]. Some studies choose to compress AP information via different methods instead of selecting AP subsets, see e.g., [17,23,24]. AP selection can be done either in the online positioning phase [24,25,27], in the offline training phase [4,26,30], or to take into account both phases [21,28]. In [28], a discrimination index is calculated for each AP in the offline phase, but the selection is performed only in the online phase, based both on discrimination index and RSS. In this study, only offline selection is included. Some older research pertaining to AP deployment, which is slightly related to our work in here, can be found also in [31,32]. In [31], location sensors are included to the positioning system, and a novel medium access scheme is proposed to control the communications between the location sensors and AP. In our study, the positioning is based only on the existing AP network, and the process is passive; hence, no communication is needed between the mobile and the AP, and, therefore, the study in [31] is out-of-scope of our research. The study in [32] focuses on the optimization of the distribution of location sensors that can also be seen as a new research topic and therefore is not considered in our study.

Youssef *et al*. [4] present the so-called max-mean method, where APs are sorted in descending order and only the strongest APs are selected, based on the maximum average RSSs. This method can be performed both in online and offline phases. In [30], Chen *et al*. suggest a novel Info Gain-strategy that is based on some AP-specific power, derived via information entropy. These two methods, often seen as traditional AP selection strategies, are considered also in our paper. The authors in [26] propose a new selection algorithm, that is based on Info Gain [30], but takes into account also possible correlation between APs. The biggest challenge with algorithms that try to minimize the correlation between selected APs [26,27] is that the correlation has to be calculated for each AP pair, leading to a huge information matrix to be handled. The correlation minimizing theme is included in our study via dissimilarity criteria and Multiple Input Multiple Output (MIMO) removal. In [29], Liang *et al*. propose a novel localization process for both offline and online phases that also utilizes AP selection based on the algorithm presented in [26]. Like in [26], the APs are chosen separately for each fingerprint in [29], and, as a result, one AP may be saved in one fingerprint, but not necessarily in many others. This leads to problems if the database is later used with some other positioning algorithm that need, e.g., estimates of the AP location (like PL and WeiC approaches), e.g., with the maxRSS-method, all APs are checked only once, not separately for each fingerprint. If the AP is not considered as strong enough, it will be discarded from the entire area (*i.e.*, whole database). The main difference between our paper and existing studies [4,23,24,25,26,27,28,29,30] is that our paper addresses both the effect of AP selection and the effect of grid size, with several different removal criteria and for three different positioning approaches. Indeed, our study is based on three multi-floor buildings, while many of the studies published in the literature are limited to one building or even to one floor within a building.

## 3. Positioning Principles

All position methods included in this paper demand an offline training phase, where the data is gathered in the building of interest. The collected data samples with known locations are further used to form the radiomap for the building. Alternative methods involve, e.g., Simultaneous Localization and Mapping (SLAM) [33,34], but our focus in this paper is on two-stage estimation.

### 3.1. Offline Training Phase

In the case of the FP approach, the whole database is needed in the positioning phase. For PL and WeiC, only parts of the radiomap information are needed in the positioning phase, but these methods also need the full radiomap in order to be able to calculate the needed parameter estimates offline. If the AP locations were known, the WeiC method would not need the full radiomap at all. Since AP positions are unknown in most cases as well as in our study, AP locations need to be estimated using the complete radiomap also for WeiC.

The FPs (also called grid points) in the radiomap are created by using fixed grid resolution [35,36,37]. This means that the FPs have a grid size that is defined by the designer, typically a square box *a* m × *a* m, with a=1,5,10, *etc*., and all gathered measurements in this area belong to the same FP. Several measurements can appear to the same FP, since the measurement collection process can be performed in different days or even continuously. In our study, when a collected data sample appears in an FP that already has a saved sample, all the APs in both samples are processed. If a new AP has been detected in the incoming sample, the AP is saved to the FP data. In that case where an AP is detected both in the old and incoming measurement, instead of using the old RSS value, we use the mean or the median between the old and new RSS values.

We form the database of FPs via: ($x_{i}$, $y_{i}$, $z_{i}$, $P_{i,k}$). In here, the 3D coordinates of the FP *i* ($i=1,...,N_{fp}$) are denoted by $x_{i}$, $y_{i}$, $z_{i}$, the total number of FPs is denoted by $N_{fp}$, and the measured RSS from the *i*-th FP to the ap-th AP is denoted by $P_{i,ap}$. An AP means one MAC address, and thus, several APs can transmit from exactly the same location (e.g., as it is in the situation of a WLAN transmitter supporting several BSSIDs). These kinds of WLAN transmitters with multiple MAC addresses are here called MIMO WLANs. The MIMO terminology comes from the 802.11n standard that supports MIMO transmissions. PL and WeiC methods use this same radiomap as the FP method to estimate the AP positions $x_{ap},y_{ap},z_{ap}$ (needed for both PL and WeiC approaches) and the PL modeling parameters transmit power $P_{T_{ap}}$ and path loss coefficient $n_{ap}$ for the ap-th AP (needed for PL based positioning).

As already mentioned, PL and WeiC approaches do not need the full radiomap but only a few parameters per AP in the estimation phase. The needed parameters are calculated in the offline training phase utilizing the full radiomap, and transferred to the mobile when needed. The most common path-loss model is the one-slope model [9]:

(1) $$P_{i,ap}\;=\;P_{T_{ap}}\;-\;10\;n_{ap}\;log_{10}\;d_{i,ap}\;+\;\eta_{i,ap}$$

Above, $n_{ap}$ stands for the path loss coefficient for the apth AP, $d_{i,ap}$ stands for the distance between the ap-th AP and the *i*-th measurement point (*i.e*., $d_{i,ap}=\sqrt{{\left(x_{i}-x_{ap}\right)}^{2}+{\left(y_{i}-y_{ap}\right)}^{2}+{\left(z_{i}-z_{ap}\right)}^{2}}$), $P_{i,ap}$ represents the observed RSS of the ap-th AP in the *i*th measurement point, $P_{T_{ap}}$ denotes the transmit power for the ap-th AP, and $\eta_{i,ap}$ is a noise factor with Gaussian distribution and standard deviation *σ* and zero mean. The one-slope PL model in Equation (1) in matrix form is [10]

(2) $$P_{\mathrm{ap}}\;=\;H_{\mathrm{ap}}\;\Theta_{\mathrm{ap}}^{T}+n$$

where $\Theta_{ap}$ contains the unknown PL parameters excluding the coordinates (*i.e*., $\Theta_{ap}=\left[n_{ap}\;P_{T_{ap}}\right]$), ${}_{T}$ is the transpose operator, n is a noise vector with Gaussian distribution and with size $N_{fp}x1$, $P_{\mathrm{ap}}$ includes the RSSs of apth AP in vector form (*i.e*., $P_{\mathrm{ap}}=\left[P_{1,ap}\;P_{2,ap}\;\cdots \;P_{N_{fp,ap},ap}\right]$), and

(3) $$H_{\mathrm{ap}}=\left[\begin{array}{cc} 1 & -10log_{10}d_{1,ap}\\ & \cdots \\ 1 & -10log_{10}d_{N_{fp,ap},ap} \end{array}\right]$$

Equation (3) can be solved through classical deconvolution approaches, such as Least Squares [9,10].

Further on, AP positions that are needed for both PL and WeiC approaches, are calculated as a weighted average over the FP positions where each AP is heard, weighted by the RSS saved in each fingerprint:

(4) $$x_{\text{ap}}=\frac{\sum_{i=1}^{N_{fp,ap}}r_{i,ap}x_{i}}{\sum_{i=1}^{N_{fp,ap}}r_{i,ap}}$$

Here, $N_{fp,ap}$ is the number of FPs where the apth AP is heard and $r_{i,ap}$ is the RSS in linear scale, *i.e*., $r_{i,ap}=10\frac{P_{i,ap}}{10}$

### 3.2. Online Estimation Phase

#### 3.2.1. Fingerprinting

Fingerprinting is a map matching localization approach, where the user location is estimated via some pattern matching algorithm using only the radiomap information together with the real-time observed RSS levels [8]. When comparing the RSSs ($O_{ap}$) observed by the user with the RSSs saved in the radiomap FPs, Bayesian estimation with Gaussian likelihood $L_{i}$ gives an estimate of user location [38]:

(5) $$L_{FP,i}\;=\;\sum_{ap=1}^{N}\;log(\frac{1}{\sqrt{2\pi \sigma_{ap}^{2}}}exp(-\frac{{\left(O_{ap}-P_{i,ap}\right)}^{2}}{2\sigma_{ap}^{2}}))$$

Here, $\sigma_{ap}$ is noise variance, which includes a shadowing component and a measurement error component and *N* is the number of APs which are heard both in the FP and in the current measurement. If no prior knowledge about $\sigma_{ap}^{2}$ is known, a fixed value can be used for all APs. Examples of typical values can be found, e.g., in [39,40]. The use of nearest neighbor (NN) averaging is also possible: $N_{n}$ FPs ${\hat{i}}_{n}$ with the maximum value of the Gaussian likelihoods $L_{{\hat{i}}_{n}}$ are selected, and the location of the MS is computed by taking the mean over the positions of $N_{n}$ nearest neighbors.

#### 3.2.2. Path Loss-Based Positioning

In the estimation phase of the PL method, the MS location is calculated using the current RSS observation $O_{ap}$ by the MS and the PL parameter estimates saved for every AP detected in the training phase (*i.e*., AP location coordinates $x_{ap}$, $y_{ap}$, $z_{ap}$, path loss coefficient $n_{ap}$ and transmit power $P_{T_{ap}}$). One approach for positioning using the path loss parameters is trilateration [41,42], where range estimates between the transmitter and the mobile are employed to estimate the position of the user. Another possible approach that is also used in this paper is to re-generate the radiomap by calculating approximate RSS levels for each heard AP for every fingerprint based on the path loss parameters sent to the MS. Further on, the position estimate can be calculated using this re-generated grid via Bayesian estimation with Gaussian likelihood, similarly as in fingerprinting:

(6) $$L_{PL,i}\;=\;\sum_{ap=1}^{N}\;log(\frac{1}{\sqrt{2\pi \sigma_{ap}^{2}}}exp(-\frac{{\left(O_{ap}-{\hat{P}}_{i,ap}\right)}^{2}}{2\sigma_{ap}^{2}}))$$

where ${\hat{P}}_{i,ap}$ presents the re-generated approximated RSS level for AP ap in fingerprint *i*.

#### 3.2.3. Weighted Centroid-Based Positioning

In the WeiC-based positioning, all that is needed in the estimation phase is the measured RSS $O_{ap}$ by the mobile and the estimated AP positions $x_{\mathrm{ap}}$ that are calculated in the training phase. Further on, the user position is calculated as an average with certain weighting factors of the positions of the heard APs in the current user measurement, weighted by their RSS [11,12]:

(7) $$x_{\mathrm{MS}}=\frac{\sum_{k=1}^{N_{ap,h}}O_{ap}x_{\mathrm{ap}}}{\sum_{k=1}^{N_{AP,h}}w_{ap}}$$

where $N_{ap,h}$ is the number of heard APs and $w_{ap}$ is the measured RSS of AP ap by the MS $O_{ap}$ in linear scale (*i.e*., $w_{ap}=10\frac{O_{ap}}{10}$).

## 4. AP Selection Criteria

In what follows, we study seven AP selection criteria in the offline training phase for all three positioning algorithms. Six out of seven have been also studied in [22], Fast Fourier transform (FFT) is a new criterion added in the comparison. Both traditional methods, max-mean [4] and InfoGain [30] are taken into account. In addition, the impact of the grid size is also studied in here. The criteria are:

- *No Selection*. In this criterion, no selection is performed, but all APs are kept in the position calculation. The criterion offers the benchmark results to understand better the effect of AP selection.
- *Maximum RSS (maxRSS)*. In this criterion, APs with maximum RSS value are selected to the AP subset. This method is basically the same as max-mean of Youssef *et al*. in [4], but the difference is that we consider only the maximum RSS instead of the average RSS when sorting the APs. The reason for this is that it has been noticed that maxRSS has similar or slightly better performance than the max-mean algorithm [21].
- *Entropy/InfoGain*. In this criterion, the so-called entropy of RSS is calculated for each AP, and the APs with maximum entropy are chosen for the AP subset. This criterion is based on InfoGain in [30], with only slight modifications. The entropy used in this paper is defined in the following manner, by using an analogy with the definition of the classical entropy [43]:
  (8) $$E_{ap}=max\;\left(P_{ap}\;\times \;log_{2}\;\left(P_{ap}\right)\right)$$
  where $P_{ap}$ includes the RSSs of apth AP in vector form (*i.e*., $P_{ap}=\left[P_{1,ap}\;P_{2,ap}\;\cdots \;P_{N_{fp,ap},ap}\right]$) and × represents the point multiplication.
- *MIMO/Multiple BSSID Selection*. As already discussed, several BSSIDs per one WLAN AP (*i.e*., transmissions with multiple MACs coming from the same physical location), are possible today. The purpose of this selection criterion is to avoid to use correlated data that the similar or closely located APs may offer. These kind of MIMO APs (or any other APs containing several MACs) are in our research identified only based on their estimated position: if more than one AP seem to be located within maximum one meter range, only one among them is chosen. The unknown AP locations are estimated as in Equation (4). Since AP infrastructure is optimized primarily for communication purposes, it is possible that two or more independent APs are really located close to each other. However, since the range-based position estimation is the only possibility in our research to define the APs that are located to each other, these kind of situations are not considered. Three different selection options are studied here: only one (with the maximum average RSS), two or three APs among the closely located ones will be kept, removal percentage being naturally the highest in the first case, where only one AP is selected. We remark that the total number of APs located at the same place (*i.e*., MIMO APs) depends on the AP infrastructure of the building.
- *FFT*. In an FFT-based criterion, we first sort the APs in a descending order, based on the spectral information computed in the FFT domain. The FFT is calculated over a matrix that contains the RSS information of each AP in every fingerprint (*i.e*., size of the matrix is $N_{ap}\times N_{fp}$). If an AP is heard in a fingerprint *i*, the RSS input is $P_{i,ap}+P_{th}$. $P_{th}$ is a threshold chosen according to an assumption that the lowest expected RSS is -100 dB, and thus, $P_{th}=100$. If an AP is not heard in the particular fingerprint, the RSS input is set to 0. After the FFT is performed over the information matrix, the APs are sorted decreasingly, based on the maximum value in the FFT-matrix.
- *KL*. In this criterion, a divergence value is calculated for every AP. This is done using the Kullback–Leibler (KL) criterion for divergence. By KL analogy, we define $D_{KL}={\left[d_{ap_{i},ap_{j}}\right]}_{ap_{i},ap_{j}=1..N_{ap}}$, where
  (9) $$d_{ap_{i},ap_{j}}=\sum_{i}\sum_{j}|P_{ap_{i}}-P_{ap_{j}}\left|\;log(\right|P_{ap_{i}}-P_{ap_{j}}\left|\right)$$
  The APs with highest KL divergence value are selected to the AP subset.
- *Dissimilarities.* Another possible criterion is based on dissimilarities between APs. First, a dissimilarity matrix is built based on the RSS differences between any pair of APs, as below:
  (10) $$D_{\mathrm{Diss}}=\left[\begin{array}{cccc} 0 & |{\bar{P}}_{1}-{\bar{P}}_{2}| & \cdots & |{\bar{P}}_{1}-{\bar{P}}_{N_{ap}}|\\ |{\bar{P}}_{2}-{\bar{P}}_{1}| & 0 & \cdots & |{\bar{P}}_{2}-{\bar{P}}_{N_{ap}}|\\ & \cdots & \cdots & \\ |{\bar{P}}_{N_{ap}}-{\bar{P}}_{1}| & |{\bar{P}}_{N_{ap}}-{\bar{P}}_{2}| & \cdots & 0 \end{array}\right]$$
  where ${\bar{P}}_{a}p$ is the mean RSS heard from ap-th AP. Further on, an independent dissimilarity value is calculated for every AP as a sum over the dissimilarities between the AP and other APs. AP subset is then chosen according to their maximum dissimilarity value.

With all selection criteria excluding MIMO selection, any removal percentage can be used: e.g., 10%, 15%, or 60% out of all APs can be removed from the radiomap database, *i.e*., the removal percentage can be flexibly chosen by the user. In MIMO selection, however, the number of co-located APs depends on the building: the Authors use the AP infrastructure as it is, and, therefore, it varies according to the building how many APs are located close to each other. Therefore, the choice of how many APs are also removed in a MIMO removal criterion depends on the building.

## 5. Measurement Analysis

### 5.1. Measurement Scenarios

**Figure 1:**
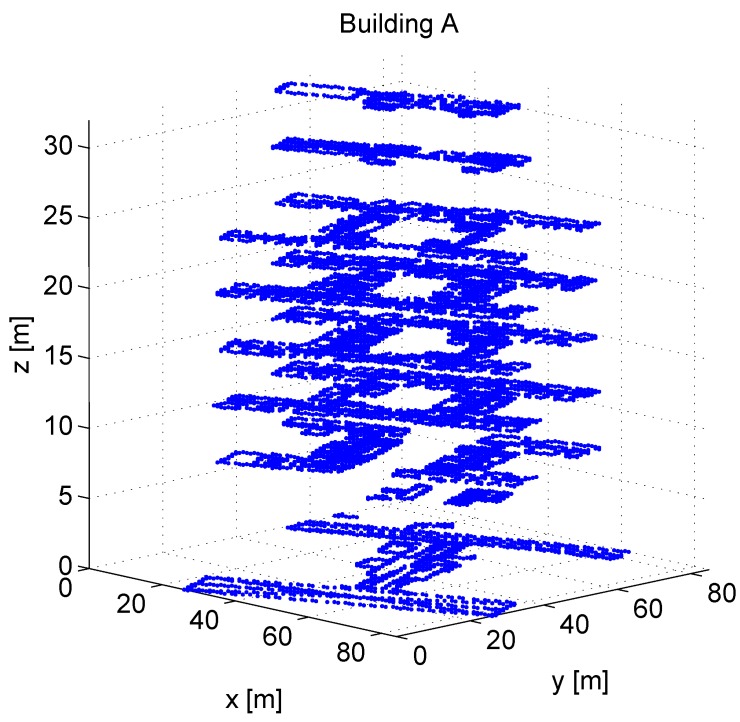
The grid of collected measurements for building A. Fixed grid resolution of 1 m ×1 m.

We collected the measurements in two multi-storey office buildings (building A located in Tampere, Finland and building B one in Berlin, Germany and one multi-floor shopping mall (*i.e*., building C located in Tampere, Finland). Figure 1 shows the FPs for building A with 1 m horizontal grid size. Measurement samples for both positioning phases (*i.e*., training and estimation phases) were gathered manually using a tablet Asus Nexus 7 with Android 4.3.1 Operating system in Tampere, Finland and in Berlin, Germany. The tablet included detailed building maps. After the training data was collected, the estimation tracks (*i.e*., the user tracks for the estimation phase) were gathered separately during different days. The measurement tracks include 250 measurements and cover all floors in all buildings. All three measurement scenarios, including building descriptions and main characteristics, are described in Table 1, showing the building location, the number of floors $N_{floors}$, the total number of detected APs saved in the radio map $N_{AP}$, number of FPs $N_{fp}$ in the radiomap with different horizontal grid resolution (here, 20 m, 10 m, 5 m and 1 m), and the number of data samples in the used user track $N_{u}$. We remark that the number of FPs $N_{fp}$ is not the same as the number of gathered measurements in the building of interest, but the number of FPs with fixed grid resolution (thus, $N_{fp}$ is dependent on the chosen resolution). The number of APs shows the amount of individual MAC addresses detected during the measurements, but, since some WLAN transmitters may have several MAC addresses due to the multiple BSSID support, some APs here may be physically at the same location.

### 5.2. Positioning Algorithm Comparison

Table 2 shows the total number of parameters needed to be stored for the training radiomap for all three positioning methods. All three buildings are included as examples with numeric values, since the total number of parameters is naturally building dependent. Besides the building size and layout, the measurement collection (*i.e*., if the whole building is covered or not) and the number of detected APs also affect the total number of parameters. For FP, the number of parameters is the sum of parameters per fingerprint, *i.e*., the fingerprint coordinates ($x_{i},y_{i},z_{i}$) and the AP index ap and the measured power for the ap-th AP $P_{i,ap}$ for each hearable AP. Since the number of heard APs may vary from one fingerprint to another, the number of parameters may be different for different fingerprints. Indeed, the parameters needed for FP positioning is remarkably decreased, if the grid size is increased, and, thus, the number of fingerprints is smaller, as can be seen in Table 2. In the case of PL and WeiC positioning approaches, the number of parameters is not dependent on grid size. For PL, all we need is the AP positions ($x_{ap},y_{ap},z_{ap}$) for each AP, together with AP dependent PL parameter estimates (here, transmit power $P_{T_{ap}}$ and path loss coefficient $n_{ap}$). For WeiC, the number of parameters is even less, namely the AP positions only. It can easily be seen, that the motivation for PL and WeiC approaches is in the low amount of data needed to be stored and transmitted. In the FP method, the number of FPs may be very large and the data that we save for each FP usually contains more than ten variables, e.g., if in a certain FP *i* the number of heard APs is 18, we need to save 39 parameters for this one fingerprint only: three parameters for the FP coordinates, 18 for RSSs and 18 for the AP indexes. However, in order to be able to calculate the AP positions and PL parameter estimates, the full radiomap is still needed for PL and WeiC approaches as well, though the amount of transmitted data is remarkably decreased.

Different positioning methods are compared in terms of time consumption in Table 3 for buildings A and B. The time is calculated over all 250 user measurements. Both 1 m and 5 m horizontal grid sizes are included, with no AP selection and with 50% AP removal. It can be seen that with 1 m grid, the FP method is clearly slower than other methods, due to a more complex pattern matching algorithm and big data matrices in the radiomap. However, when the grid size is increased to 5 m, the time consumption for FP is decreased remarkably, even more than 80%. Indeed, the difference between the FP and PL methods is clearly smaller with the 5 m grid. For the PL approach, 50% AP removal slightly decreases the time consumption. WeiC algorithm, that uses only AP positions in the estimation, is not affected by the decrease in the number of APs. Naturally, the grid size also does not affect the time consumption of PL and WeiC methods at all, since the number of parameters for these methods remains the same for all grid sizes, as seen in Table 2.

### 5.3. AP Selection

The localization results for AP selection are presented in this section as Mean Distance Error (MDE) in 3D. MDE is calculated by taking the main of the Euclidean distances between the user location estimates and the true user locations in a 3D (x,y,z) Cartesian coordinate system. For the FP method, the NN-method is also used, with $N_{n}=5$.

**Figure 2:**
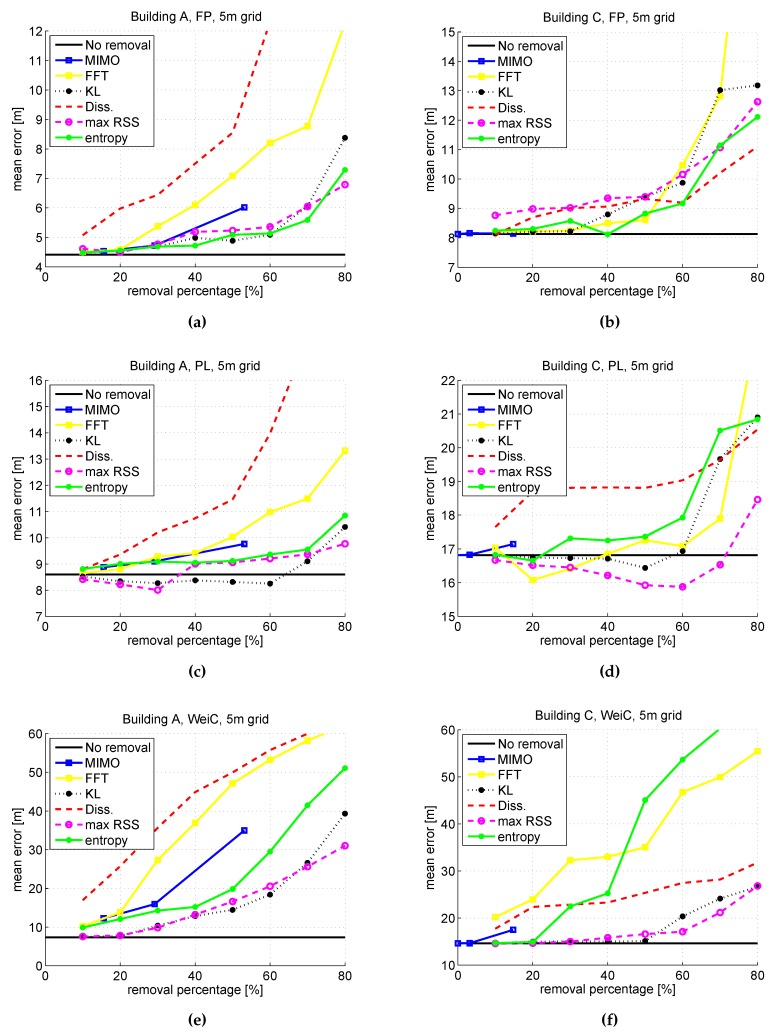
Average positioning error [m] for all AP selection criteria. FP, PL and WeiC positioning approaches. Building A (**a**,**c**,**e**) and building C (**b**,**d**,**f**) with 5 m horizontal grid size.

Figure 2 shows the MDE for the seven investigated AP selection criteria for buildings A and C, with all three positioning approaches and 5 m horizontal grid size. The percentage of removed APs from the radiomap varies between 10% and 80%, excluding the MIMO case. In the case of MIMO selection, the number of removed APs is varied so that either one (with the maximum average RSS value), two or three APs were kept out of the APs located next to each other (within 1 m range). When we keep only one AP, the removal percentage is the highest compared to the case when we remove two or three APs. It can easily be seen in Figure 2a,c,e that maxRSS, KL and entropy-based selection criteria are the best choices in building A, with no big difference to each other. The results are similar to every positioning method. Indeed, removing APs using MIMO AP selection criteria and and keeping only one AP among the subset of co-located APs does not increase the positioning error. This however holds only for FP and PL approaches, and it should be noticed, that since the number of MIMO APs is building dependent, the percentage of removed APs may vary between only a few percent and even 60%. This is the case also in building C (Figure 2b,d,f that has clearly less detected APs in the radiomap database (as can be seen in Table 1), and the MIMO selection criterion removes only 17% of the APs. When it comes to the other criteria for building C, maxRSS is also the best criterion in this building in the case of PL and WeiC, but for the FP method, the differences between different criteria are smaller. In general, WeiC positioning seems to be very sensitive to AP selection, and basically only 20% of the APs can be removed safely without deteriorating the results. In the case of FP, APs can be removed up to 50%--60%, but after that, the performance starts to decrease faster. The PL method instead is also very robust to high percentage removals. Based both on the Figure 2 and also on the results obtained for building B and our previous studies in [21,22], it is observed that the best results are obtained with maxRSS-based AP removal criterion. This criterion is also most consistent among all criteria in general, if 50% of the APs are removed from the radiomap database. This holds for both FP and PL positioning methods, but for WeiC, the AP selection should be performed with much lower removal percentages, if any.

**Figure 3:**
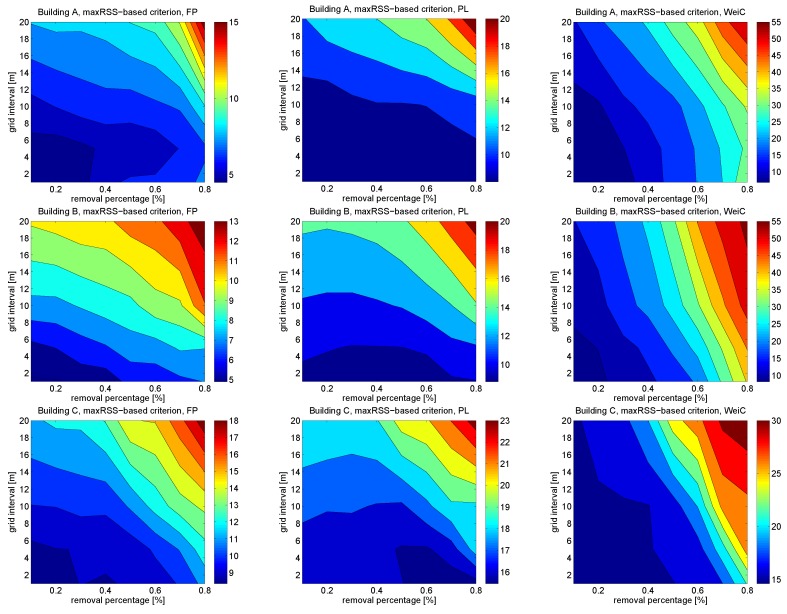
Effects of AP removal and grid size for buildings A, B and C. FP, PL and WeiC positioning positioning approaches and maxRSS-selection criterion.

Figure 3 illustrates the effects of AP removal and grid size, for all three positioning approaches and maxRSS-selection criterion. The grid size is varied between 1 m and 20 m and the removal percentage between 10% and 80%. We remark that the color bars are different for each figure. The reason for this is that the results vary in the case of WeiC so widely (between 10 m and 55 m) that the much smaller variation for FP (between 5 m and 15 m) would not be visible anymore if the same color bar was used for all figures. Also in Figure 3, it can be seen that the WeiC method is very sensitive to AP removal, but the grid size affects less. On the other hand, as it was noticed already in Figure 2, the PL method is robust to AP removal also with high removal percentages, but the grid size affects more. This is the case also for fingerprinting, though the positioning accuracy for FP in general is better than for PL, e.g., for 50% removal and 10 m grid, the positioning accuracy is around 9 m for FP and 12 m for PL (building A), 7 m for FP and 10 m for PL (building B) and 11 m for FP and 17 m for PL (building C). We remark that both the FP and PL methods still achieve reasonable results, with as high as 10 m grids, and 50% of the APs are removed, especially in buildings A and B that have more detected APs than building C.

## 6. Conclusions

In this paper, three positioning approaches, namely FP, PL and WeiC, have been compared in terms of complexity and performance. All results are based on real field measurements in three multi-floor office buildings with thousands of gathered fingerprints. Indeed, the effects of grid size and AP reduction to different methods is studied. We have validated our earlier findings concerning several different AP selection criteria, including one new proposed criterion, and concluded that the maxRSS-based removal criterion gives consistently the best results in the majority of scenarios. We have shown that in the case of FP, 50% of the APs can be removed safely from the training database with a properly chosen removal criterion without deteriorating the positioning results much. Indeed, we have shown that the low-complexity method WeiC is very sensitive to AP reduction, while the PL method is also very robust to high percentage removals. If the grid size is increased to 5 or even 10 m, the performance for every positioning method remains tolerable, but the number of parameters in the radiomap needed especially for the FP method can be significantly decreased. Our study is most useful in mobile-centric approaches but is valid also in the network-centric cases.

## Figures and Tables

**Table 1:** Measurement scenarios. $N_{fp}$ corresponds to the number of fingerprints with fixed grid resolution in the database.

	Location	Grid Resolution	$N_{fp}$	$N_{u}$	$N_{AP}$	$N_{floors}$
A	Berlin, Germany	1 m	14,611	250	727	9
		5 m	1446			
		10 m	516			
B	Tampere, Finland	1 m	8201	250	1213	4
		5 m	1082			
		10 m	398			
C	Tampere, Finland	1 m	1988	250	162	3
		5 m	373			
		10 m	141			

**Table 2:** Number of the parameters needed to be transmitted for different positioning methods. Examples for buildings A and B.

		FP	PL	WeiC
		$\sum_{n=1}^{N_{FP}}\left(3+\sum_{m=1}^{N_{AP_{FP}}}2\right)$	$N_{AP}\times 5$	$N_{AP}\times 3$
Building A	1 m grid	$N_{A_{1}}=1,091,029$	3635 (≈0.3% of $N_{A_{1}}$)	2181 (≈0.2% of $N_{A_{1}}$)
	5 m grid	$N_{A_{2}}=171,090$	3635 (≈2.1% of $N_{A_{2}}$)	2181 (≈1.3% of $N_{A_{2}}$)
	10 m grid	$N_{A_{3}}=76,074$	3635 (≈4.8% of $N_{A_{3}}$)	2181 (≈2.9% of $N_{A_{3}}$)
Building B	1 m grid	$N_{B_{1}}=1,196,629$	6065 (≈0.5% of $N_{B_{1}}$)	3639 (≈0.3% of $N_{B_{1}}$)
	5 m grid	$N_{B_{2}}=225,246$	6065 (≈2.7% of $N_{B_{2}}$)	3639 (≈1.6% of $N_{B_{2}}$)
	10 m grid	$N_{B_{3}}=104,682$	6065 (≈5.8% of $N_{B_{3}}$)	3639 (≈3.5% of $N_{B_{3}}$)
Building C	1 m grid	$N_{C_{1}}=90,542$	810 (≈0.9% of $N_{C_{1}}$)	486 (≈0.5% of $N_{C_{1}}$)
	5 m grid	$N_{C_{2}}=23,025$	810 (≈3.5% of $N_{C_{2}}$)	486 (≈2.1% of $N_{C_{2}}$)
	10 m grid	$N_{C_{3}}=10,483$	810 (≈7.7% of $N_{C_{3}}$)	486 (≈4.6% of $N_{C_{3}}$)

**Table 3:** Performance comparison via algorithm time consumption [s] for 250 user measurements. All positioning methods included with 1 m and 5 m grids, with no AP selection and 50% AP removal.

Building	Grid [m]		FP	PL	WeiC
Building A	1	No removal	$t_{1}=1483$ s	$0.10\times t_{1}$	$1.2\times 10^{-5}\times t_{1}$
		50% removal	$0.81\times t_{1}$	$0.07\times t_{1}$	$1.2\times 10^{-5}\times t_{1}$
	5	No removal	$0.18\times t_{1}$	$0.10\times t_{1}$	$1.2\times 10^{-5}\times t_{1}$
		50% removal	$0.17\times t_{1}$	$0.06\times t_{1}$	$1.2\times 10^{-5}\times t_{1}$
Building B	1	No removal	$t_{2}=2472.0$ s	$0.08\times t_{2}$	$7.8\times 10^{-6}\times t_{2}$
		50% removal	$0.82\times t_{2}$	$0.06\times t_{2}$	$7.8\times 10^{-6}\times t_{2}$
	5	No removal	$0.23\times t_{2}$	$0.08\times t_{2}$	$7.8\times 10^{-6}\times t_{2}$
		50% removal	$0.13\times t_{2}$	$0.05\times t_{2}$	$7.8\times 10^{-6}\times t_{2}$
